# Supplementary material for: Accelerated nitrogen cycling on Mediterranean seagrass leaves at volcanic CO2 vents
Source: Commun Biol. 2024 Mar 19;7:341. doi: 10.1038/s42003-024-06011-0 (PMC11254932; doi:10.1038/s42003-024-06011-0)
Supplement: Supplementary file 2 — Supplementary Information [file 42003_2024_6011_MOESM2_ESM.pdf]

Supplementary material

Accelerated Nitrogen Cycling on Mediterranean seagrass leaves at volcanic CO_2 vents

Authors

Johanna Berlinghof, Luis M. Montilla, Friederike Peiffer, Grazia M. Quero, Ugo Marzocchi, Travis B. Meador, Francesca Margiotta, Maria Abagnale, Christian Wild, Ulisse Cardini

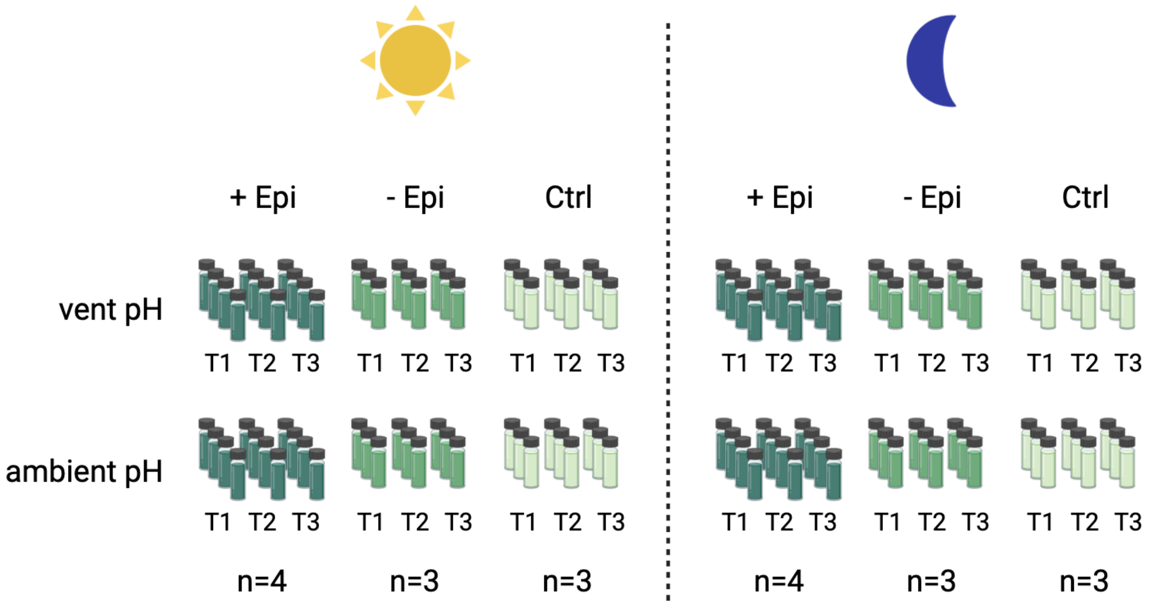

Suppl. Figure 1. Experimental design of the three isotopic tracer incubation experiments with light and dark incubations, leaves from vent and ambient pH, with epiphytes present or removed, as well as controls. T1, 2, and 3 represent samples that were opened at different timepoints.

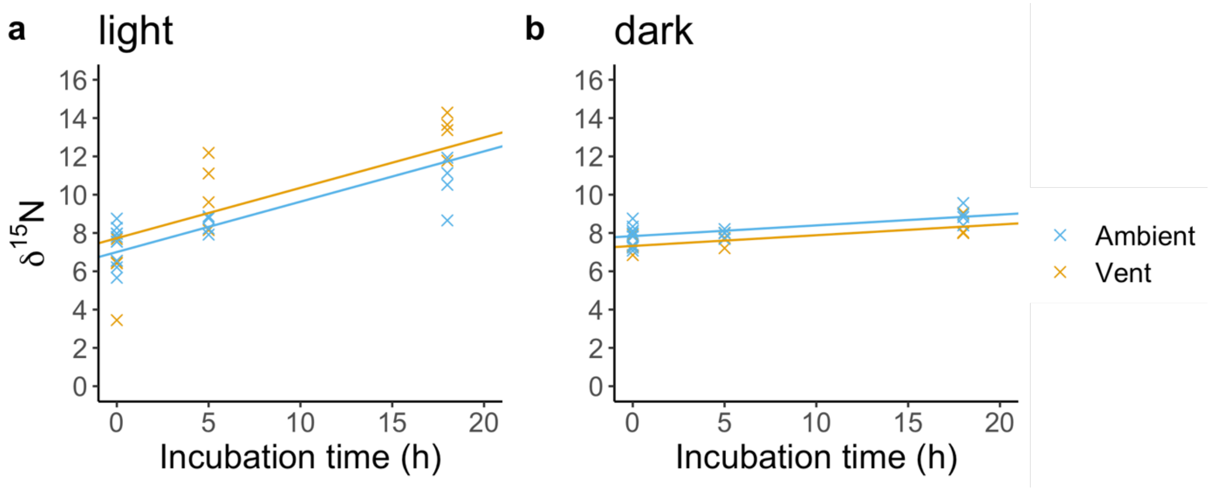

Suppl. Figure 2. δ^{15}N increase during light (**a**) and dark (**b**) incubations in <u>epiphytes</u> from the ambient and the vent site. Solid lines represent linear regressions.

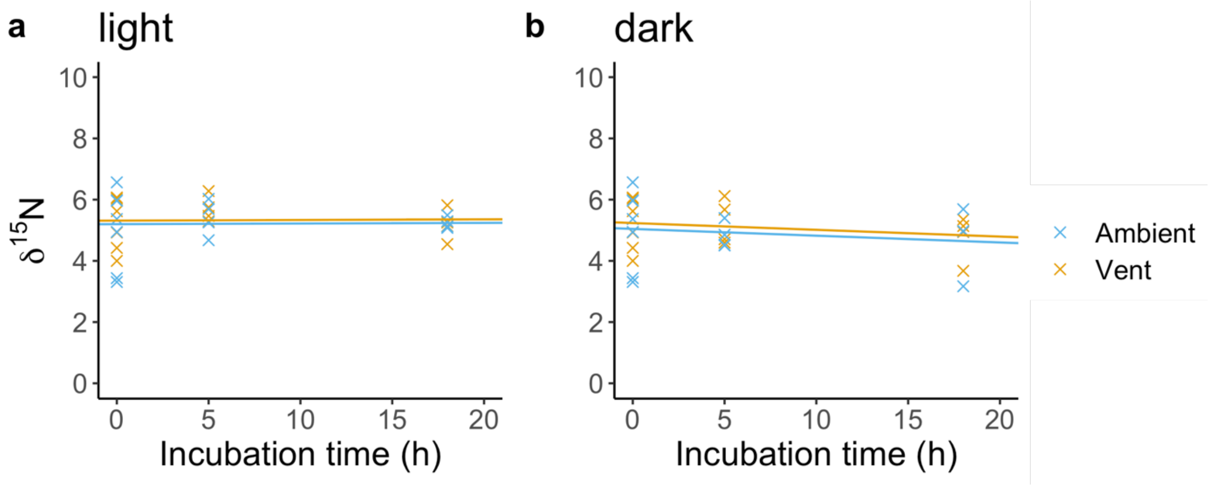

Suppl. Figure 3. δ^{15}N increase during light (**a**) and dark (**b**) incubations in <u>seagrass leaf sections</u> from the ambient and the vent site. Solid lines represent linear regressions.

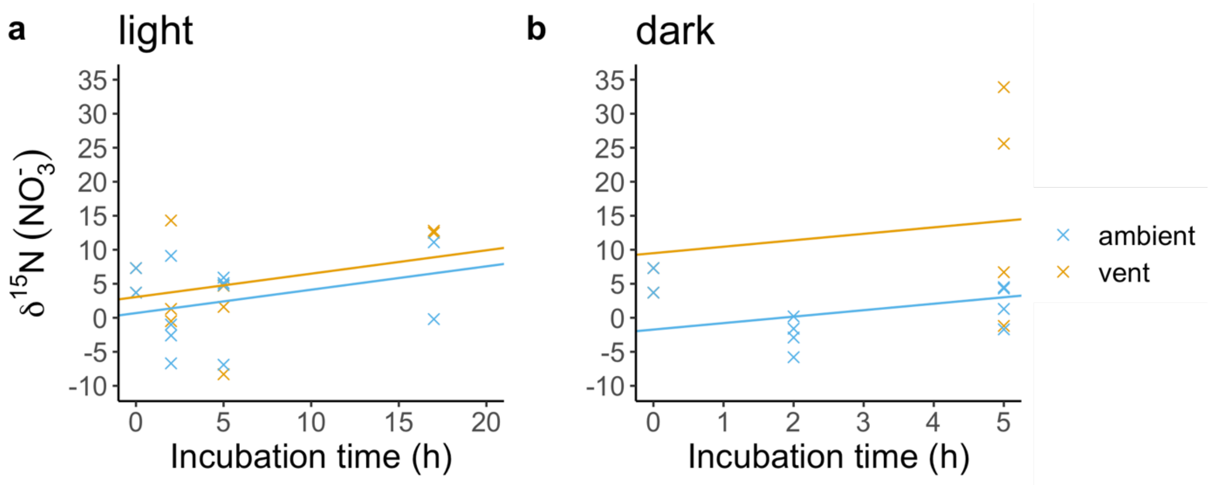

Suppl. Figure 4. δ^{15}N (NO_3^-) increase during light (a) and dark (b) incubations with seagrass leaf sections with epiphytes. Solid lines represent linear regressions.

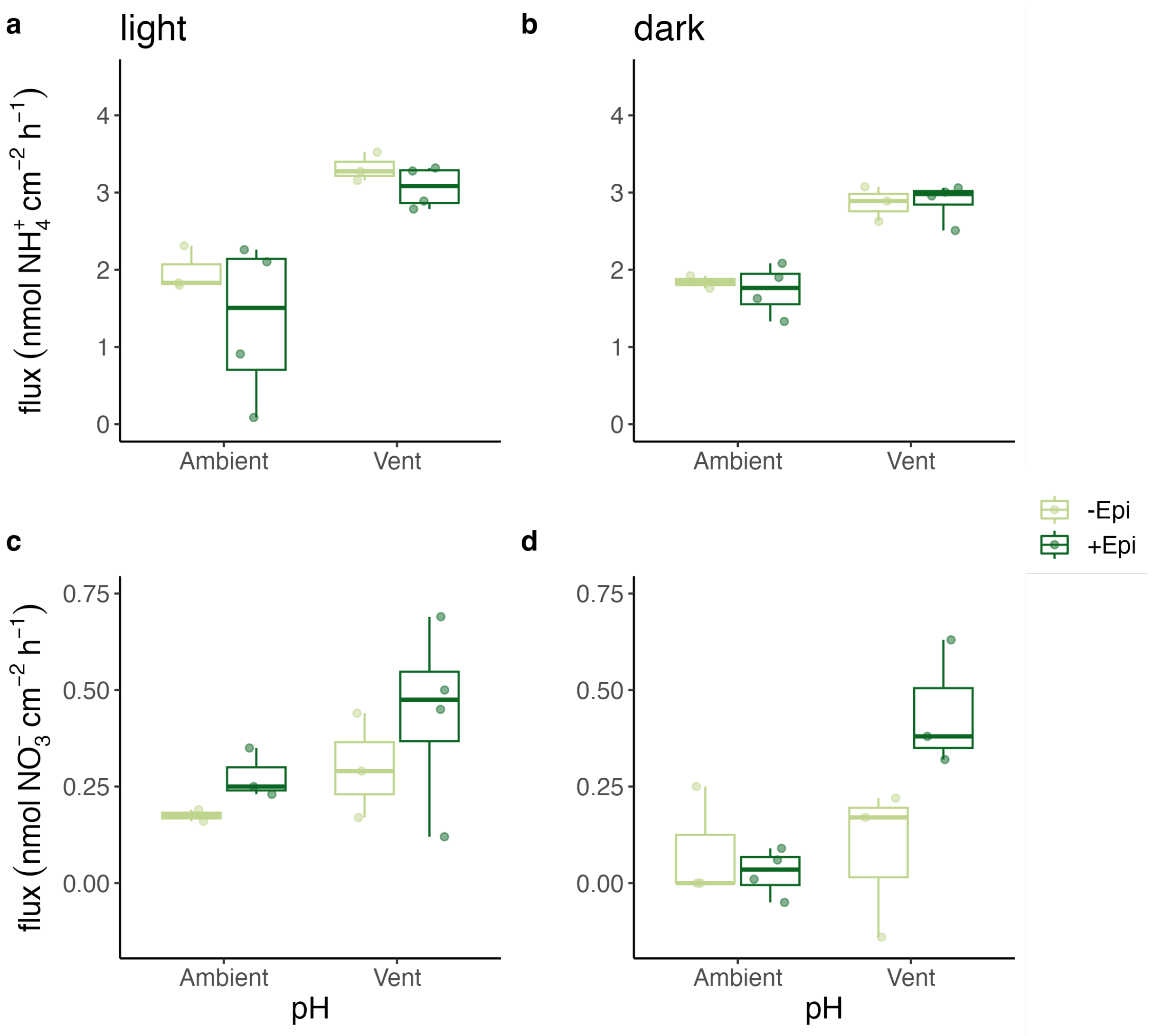

Suppl. Figure 5. Uptake of NH_4^+ (**a, b**) and NO_3^- (**c, d**) during light (**a, c**) and dark (**b, d**) incubations with leaf sections from the ambient and vent site with (+Epi, n=4) and without epiphytes (-Epi, n=3). Error bars indicate mean ± SE.

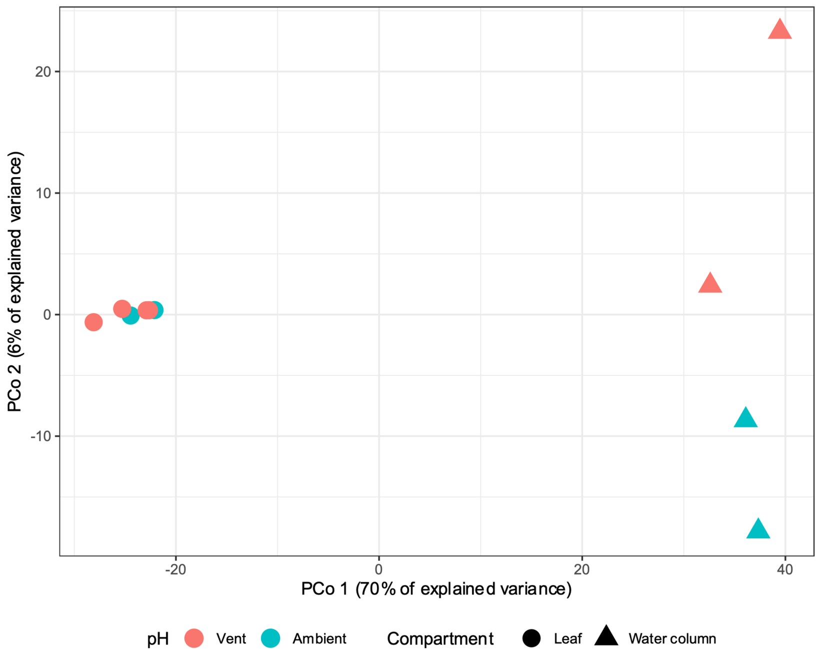

Suppl. Figure 6. Principal coordinates analysis of the prokaryote community from the leaves and water column on both pH regimes.

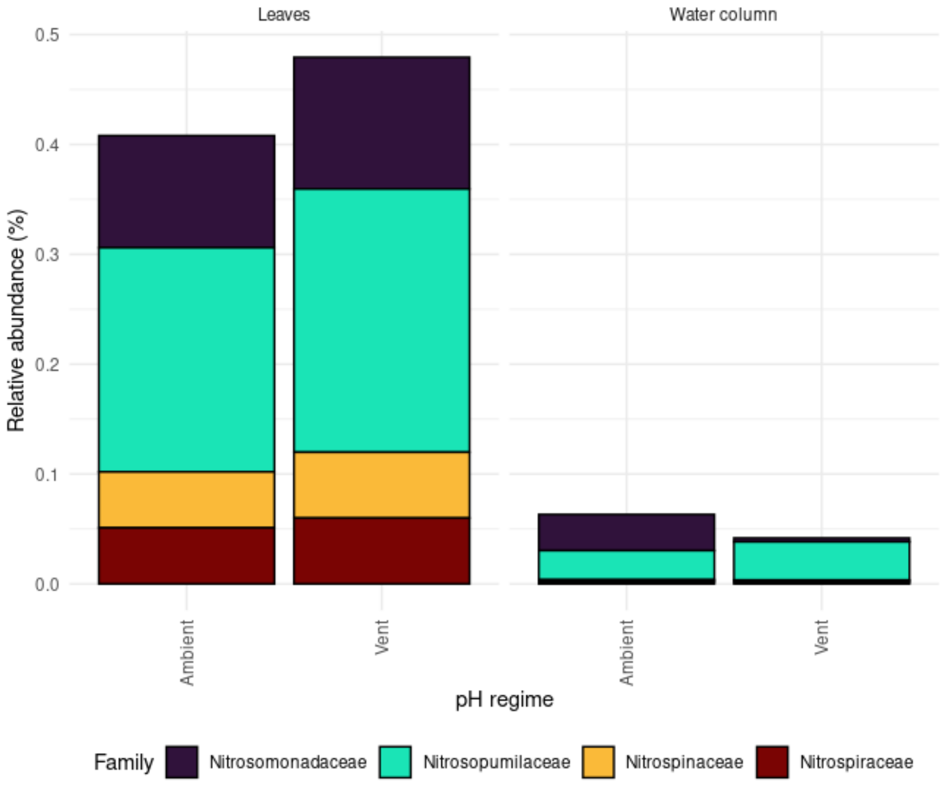

Suppl. Figure 7. Relative abundances of nitrifying prokaryotic taxa collapsed at the family level on leaves and water column samples from both pH regimes.

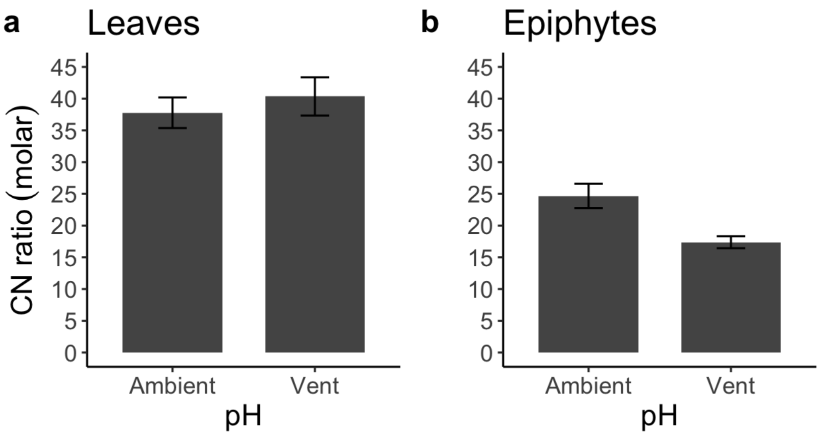

Suppl. Figure 8. C:N ratios of leaf sections (**a**) and epiphytes (**b**) from the ambient (*n* leaves = 14, *n* epiphytes =8) and vent site (*n* leaves = 14, *n* epiphytes =7). Since there were no differences between light and dark incubations, the samples were combined and treated as replicates. Error bars indicate mean ± SE.

Suppl. Table 1. Permutation-based analysis of variance of the microbial communities associated with *P. oceanica* leaves, water column and pH regime.

Source of variation	Degrees of freedom	Sum of squares	R^2	Pseudo-F	P(>F)
pH regime	1	821.5626	0.0641022	2.013119	0.1885
Compartment	1	8928.5753	0.6966495	21.878170	0.0010
Treatment x Compartment	1	617.6887	0.0481950	1.513556	0.2077
Residual	6	2448.6258	0.1910533	NA	NA
Total	9	12816.4525	1.0000000	NA	NA

Suppl. Table 2. Permutation-based analysis of variance of the nitrifying communities associated with *P. oceanica* leaves, water column and pH regime.

Source of variation	Degrees of freedom	Sum of squares	R^2	Pseudo-F	P(>F)
pH regime	1	1.16E-08	0.0009	0.7777	0.9858
Compartment	1	1.44E-06	0.1125	9.6679	0.0001
Treatment x Compartment	1	2.25E-08	0.0017	0.1506	0.96
Residual	76	1.13E-05	0.8879	NA	NA
Total	79	1.28E-05	1.0000000	NA	NA

Suppl. Table 3. Environmental parameters (mean ± SE, n=3) measured at the vent and ambient pH site at Castello Aragonese.

	Ambient pH	Vent pH
T (°C)	23.94 ± 0.05	23.74 ± 0.01
Light (Lux)	10438 ± 872	16631 ± 628
pH	8.07 ± 0.08	7.06 ± 0.37
DO (mg L^{-1})	9.15 ± 0.02	8.26 ± 0.02

Average temperature, light, and DO were continuously measured with data loggers during the sampling time between 11 am and 4 pm of the respective sampling day. PH was measured on 13.09.2019 with a pH logger (n ambient = 15, n vent = 8)

Suppl. Table 4. $^{29}N_2$ and $^{30}N_2$ concentrations in the denitrification experiment at different incubation timepoints (mean ± SD).

Site	Timepoint	Incubation	Treatment	$^{29}N_2$ concentration (nmol/L)	$^{30}N_2$ concentration (nmol/L)
Vent	T0			0.080 ± 0.015	0.263 ± 0.012
Vent	T1	light	+Epi	0.075 ± 0.008	0.313 ± 0.024
Vent	T1	light	-Epi	0.062 ± 0.006	0.355 ± 0.013
Vent	T1	dark	+Epi	0.057 ± 0.008	0.323 ± 0.056
Vent	T1	dark	-Epi	0.056 ± 0.008	0.249 ± 0.007
Vent	T2	light	control	0.091 ± 0.018	0.019 ± 0.038
Vent	T2	dark	control	0.042 ± 0.051	-0.015 ± 0.054
Vent	T2	light	+Epi	0.144 ± 0.036	0.369 ± 0.059
Vent	T2	light	-Epi	0.105 ± 0.027	0.091 ± 0.021
Vent	T2	dark	+Epi	0.097 ± 0.013	0.069 ± 0.008
Vent	T2	dark	-Epi	0.098 ± 0.010	0.062 ± 0.012
Ambient	T0			0.059 ± 0.015	0.302 ± 0.041
Ambient	T1	light	+Epi	0.022 ± 0.080	0.231 ± 0.063
Ambient	T1	light	-Epi	0.010 ± 0.114	0.229 ± 0.060
Ambient	T1	dark	+Epi	0.025 ± 0.067	0.206 ± 0.062
Ambient	T1	dark	-Epi	-0.006 ± 0.096	0.209 ± 0.009
Ambient	T2	light	control	0.104 ± 0.013	0.026 ± 0.006
Ambient	T2	dark	control	0.097 ± 0.012	0.050 ± 0.023
Ambient	T2	light	+Epi	0.122 ± 0.015	0.053 ± 0.037
Ambient	T2	light	-Epi	0.072 ± 0.051	0.036 ± 0.043
Ambient	T2	dark	+Epi	0.110 ± 0.014	0.024 ± 0.030
Ambient	T2	dark	-Epi	0.035 ± 0.048	-0.071 ± 0.104

Suppl. Table 5. Morphological traits (mean ± SE) of *P. oceanica* from ambient and vent pH sites.

	Ambient pH	Vent pH
Shoot density (m^{-2})	527.38 ± 110.90	1130.09 ± 234.24
Leaf density (m^{-2})	4237.85 ± 515.01	7496.29 ± 674.21
Leaf dry weight (g)	0.041 ± 0.005	0.087 ± 0.013
