# Supplementary material for: Accelerated nitrogen cycling on Mediterranean seagrass leaves at volcanic CO2 vents
Source: Commun Biol. 2024 Mar 19;7:341. doi: 10.1038/s42003-024-06011-0 (PMC11254932; doi:10.1038/s42003-024-06011-0)
Supplement: Supplementary file 3 — Description of Additional Supplementary Files [file 42003_2024_6011_MOESM3_ESM.pdf]

Description of Additional Supplementary Files

File name: Supplementary Data
Description: Source data for all figures and tables.
