# Supplementary material for: Accelerated nitrogen cycling on Mediterranean seagrass leaves at volcanic CO2 vents
Source: Commun Biol. 2024 Mar 19;7:341. doi: 10.1038/s42003-024-06011-0 (PMC11254932; doi:10.1038/s42003-024-06011-0)
Supplement: Supplementary file 5 — Reporting Summary [file 42003_2024_6011_MOESM5_ESM.pdf]

nature portfolio

Corresponding author(s): Johanna Berlinghof, Ulisse Cardini

Last updated by author(s): Jan 31, 2024

Reporting Summary

Nature Portfolio wishes to improve the reproducibility of the work that we publish. This form provides structure for consistency and transparency in reporting. For further information on Nature Portfolio policies, see our Editorial Policies and the Editorial Policy Checklist.

Statistics

For all statistical analyses, confirm that the following items are present in the figure legend, table legend, main text, or Methods section.

n/a	Confirmed	
☐	☒	The exact sample size (*n*) for each experimental group/condition, given as a discrete number and unit of measurement
☐	☒	A statement on whether measurements were taken from distinct samples or whether the same sample was measured repeatedly
☐	☒	The statistical test(s) used AND whether they are one- or two-sided<br>*Only common tests should be described solely by name; describe more complex techniques in the Methods section.*
☐	☒	A description of all covariates tested
☐	☒	A description of any assumptions or corrections, such as tests of normality and adjustment for multiple comparisons
☐	☒	A full description of the statistical parameters including central tendency (e.g. means) or other basic estimates (e.g. regression coefficient) AND variation (e.g. standard deviation) or associated estimates of uncertainty (e.g. confidence intervals)
☐	☒	For null hypothesis testing, the test statistic (e.g. *F*, *t*, *r*) with confidence intervals, effect sizes, degrees of freedom and *P* value noted<br>*Give P values as exact values whenever suitable.*
☒	☐	For Bayesian analysis, information on the choice of priors and Markov chain Monte Carlo settings
☐	☒	For hierarchical and complex designs, identification of the appropriate level for tests and full reporting of outcomes
☒	☐	Estimates of effect sizes (e.g. Cohen's *d*, Pearson's *r*), indicating how they were calculated

Our web collection on statistics for biologists contains articles on many of the points above.

Software and code

Policy information about availability of computer code

Data collection	No software was used for data collection
Data analysis	cutadapt v2.8, R(2023), R packages DADA2, vegan, ALDEX2<br>The complete pipeline is openly available in the research compendium accompanying this paper at https://github.com/luismmontilla/embrace.

For manuscripts utilizing custom algorithms or software that are central to the research but not yet described in published literature, software must be made available to editors and reviewers. We strongly encourage code deposition in a community repository (e.g. GitHub). See the Nature Portfolio guidelines for submitting code & software for further information.

Data

Policy information about availability of data

All manuscripts must include a data availability statement. This statement should provide the following information, where applicable:

- Accession codes, unique identifiers, or web links for publicly available datasets
- A description of any restrictions on data availability
- For clinical datasets or third party data, please ensure that the statement adheres to our policy

All data needed to evaluate the conclusions of the paper are provided in the paper and/or in the Supplementary Materials. Source data for all N isotope

experiments, net dissolved inorganic N fluxes and C:N ratios, and 16S rRNA gene ASV tables, are provided with this paper. Raw sequencing data supporting the results of this study have been deposited in the NCBI SRA database with the BioProject accession code: PRJNA824287. The source data are provided with this paper.

Research involving human participants, their data, or biological material

Policy information about studies with human participants or human data. See also policy information about sex, gender (identity/presentation), and sexual orientation and race, ethnicity and racism.

Reporting on sex and gender	na
Reporting on race, ethnicity, or other socially relevant groupings	na
Population characteristics	na
Recruitment	na
Ethics oversight	na

Note that full information on the approval of the study protocol must also be provided in the manuscript.

Field-specific reporting

Please select the one below that is the best fit for your research. If you are not sure, read the appropriate sections before making your selection.

☐ Life sciences ☐ Behavioural & social sciences ☒ Ecological, evolutionary & environmental sciences

For a reference copy of the document with all sections, see nature.com/documents/nr-reporting-summary-flat.pdf

Ecological, evolutionary & environmental sciences study design

All studies must disclose on these points even when the disclosure is negative.

Study description	The effects of long-term natural ocean acidification on the epiphytic prokaryotic community of Posidonia oceanica leaves were tested and rates of key nitrogen (N) cycling processes by the plant phyllosphere were quantified. The effects of pH and the presence/absence of epiphytes were tested in multifactorial laboratory incubations (vent pH vs ambient pH, light vs dark, with epiphytes vs without epiphytes), using N stable isotope tracers to quantify N2 fixation, nitrification potential, and anammox and denitrification potential, and net nutrient fluxes to quantify assimilatory processes by leaves and epiphytes. Tthese analyses were complemented with 16s rRNA gene amplicon sequencing to explore the diversity of the phyllosphere microbial community and the potential players involved in N transformation processes on seagrass leaves.
Research sample	Leaf sections and microbial community samples of P. oceanica from vent and ambient pH sites at Ischia island (Italy)
Sampling strategy	For the incubation experiments, shoots of P. oceanica were collected at each site on three days in September 2019 and transported directly to the laboratory. Sections of the central part of the leaf (3 cm in length) were cut off, selecting leaves with homogeneous epiphyte coverage, and avoiding heavily grazed and senescent parts of the plant. Epiphytes were carefully removed from half of the seagrass leaves with a scalpel, taking special care not to damage the plant tissue. Leaf sections from the vent pH and ambient pH sites, with epiphytes present (n = 4) or removed (n = 3), were used for dark and incubations to ensure sufficient replication but avoiding taking too much seagrass shoots from the meadows.<br>Samples for microbial community analysis were collected in October 2019 at the vent and ambient site described above. Before disturbing the plants, we collected 5 L of seawater from the water column above the plants. Whole seagrass plants (n=5) were collected, and the central part of the leaf was cut off with sterile tools, washed with sterile NaCl solution [0.8 % m/v] to remove loosely attached microorganisms, and transferred to 15 mL falcon tubes with sterile tweezers. The falcon tubes were kept in dry ice during transport to the laboratory (SZN Villa Dohrn, Ischia, Italy) and then stored at -20°C. In the laboratory, the seawater was immediately filtered on 0.2 µm cellulose nitrate membrane filters (n=5) and the filters were stored at -20°C until further genetic analysis.
Data collection	Field data (light, temperature, pH, oxygen) was measured with data loggers on the respective days when the experiments were carried out.
Timing and spatial scale	Experiments were performed in September 2019. The study area is located at the islet of Castello Aragonese on the northeastern coast of the island of Ischia (Tyrrhenian Sea, Italy). The vent pH site was located in a vent area on the south side (40°43'50.5"N 13°57'47.2"E) and the ambient pH site was located on the north side of the bridge (40°43'54.8"N 13°57'47.1"E).
Data exclusions	Samples of 16S sequencing were excluded, when the sequencing quality was poor
Reproducibility	Field experiments and sampling was not repeated.

Randomization	Leaf sections were grouped in vent and ambient pH and with/without epiphytes and then distributed randomly for light and dark incubations.
Blinding	Blinding was not applicable, because sample groups needed to be distinguishable for respective sample treatment (e.g. covered for dark incubations).

Did the study involve field work? ☒ Yes ☐ No

Field work, collection and transport

Field conditions	Field conditions are summarized in Suppl. Table 3
Location	The study area is located at the islet of Castello Aragonese on the northeastern coast of the island of Ischia (Tyrrhenian Sea, Italy). The vent pH site was located in a vent area on the south side (40°43'50.5"N 13°57'47.2"E) and the ambient pH site was located on the north side of the bridge (40°43'54.8"N 13°57'47.1"E).
Access & import/export	Samples were collected via scuba diving.
Disturbance	Sample size was adjusted to minimize disturbance.

Reporting for specific materials, systems and methods

We require information from authors about some types of materials, experimental systems and methods used in many studies. Here, indicate whether each material, system or method listed is relevant to your study. If you are not sure if a list item applies to your research, read the appropriate section before selecting a response.

Materials & experimental systems

n/a	Involved in the study	
☐	☐	Antibodies
☐	☐	Eukaryotic cell lines
☐	☐	Palaeontology and archaeology
☐	☐	Animals and other organisms
☐	☐	Clinical data
☐	☐	Dual use research of concern
☐	☒	Plants

Methods

n/a	Involved in the study	
☐	☐	ChIP-seq
☐	☐	Flow cytometry
☐	☐	MRI-based neuroimaging

Antibodies

Antibodies used	na
Validation	na

Eukaryotic cell lines

Policy information about cell lines and Sex and Gender in Research

Cell line source(s)	na
Authentication	na
Mycoplasma contamination	na
Commonly misidentified lines (See ICLAC register)	na

Palaeontology and Archaeology

Specimen provenance	na
Specimen deposition	na

Dating methods	na

☐ Tick this box to confirm that the raw and calibrated dates are available in the paper or in Supplementary Information.

Ethics oversight	na

Note that full information on the approval of the study protocol must also be provided in the manuscript.

Animals and other research organisms

Policy information about studies involving animals; ARRIVE guidelines recommended for reporting animal research, and Sex and Gender in Research

Laboratory animals	na
Wild animals	na
Reporting on sex	na
Field-collected samples	na
Ethics oversight	na

Note that full information on the approval of the study protocol must also be provided in the manuscript.

Clinical data

Policy information about clinical studies

All manuscripts should comply with the ICMJE guidelines for publication of clinical research and a completed CONSORT checklist must be included with all submissions.

Clinical trial registration	na
Study protocol	na
Data collection	na
Outcomes	na

Dual use research of concern

Policy information about dual use research of concern

Hazards

Could the accidental, deliberate or reckless misuse of agents or technologies generated in the work, or the application of information presented in the manuscript, pose a threat to:

No	Yes	
☒	☐	Public health
☒	☐	National security
☒	☐	Crops and/or livestock
☒	☐	Ecosystems
☒	☐	Any other significant area

Experiments of concern

Does the work involve any of these experiments of concern:

No	Yes	
☒	☐	Demonstrate how to render a vaccine ineffective
☒	☐	Confer resistance to therapeutically useful antibiotics or antiviral agents
☒	☐	Enhance the virulence of a pathogen or render a nonpathogen virulent
☒	☐	Increase transmissibility of a pathogen
☒	☐	Alter the host range of a pathogen
☒	☐	Enable evasion of diagnostic/detection modalities
☒	☐	Enable the weaponization of a biological agent or toxin
☒	☐	Any other potentially harmful combination of experiments and agents

Plants

Seed stocks	field collection of P. oceanica shoots
Novel plant genotypes	na
Authentication	na

ChIP-seq

Data deposition

☐ Confirm that both raw and final processed data have been deposited in a public database such as GEO.

☐ Confirm that you have deposited or provided access to graph files (e.g. BED files) for the called peaks.

Data access links<br>*May remain private before publication.*	na
Files in database submission	na
Genome browser session<br>(e.g. UCSC)	na

Methodology

Replicates	na
Sequencing depth	na
Antibodies	na
Peak calling parameters	na
Data quality	na
Software	na

Flow Cytometry

Plots

Confirm that:

- [ ] The axis labels state the marker and fluorochrome used (e.g. CD4-FITC).
- [ ] The axis scales are clearly visible. Include numbers along axes only for bottom left plot of group (a 'group' is an analysis of identical markers).
- [ ] All plots are contour plots with outliers or pseudocolor plots.
- [ ] A numerical value for number of cells or percentage (with statistics) is provided.

Methodology

Sample preparation	na
Instrument	na
Software	na
Cell population abundance	na
Gating strategy	na

- [ ] Tick this box to confirm that a figure exemplifying the gating strategy is provided in the Supplementary Information.

Magnetic resonance imaging

Experimental design

Design type	na
Design specifications	na
Behavioral performance measures	na

Acquisition

Imaging type(s)	na
Field strength	na
Sequence & imaging parameters	na
Area of acquisition	na

Diffusion MRI ☐ Used ☐ Not used

Preprocessing

Preprocessing software	na
Normalization	na
Normalization template	na
Noise and artifact removal	na
Volume censoring	na

Statistical modeling & inference

Model type and settings	na
Effect(s) tested	na

Specify type of analysis: ☐ Whole brain ☐ ROI-based ☐ Both

Statistic type for inference

(See Eklund et al. 2016)

na

Correction

na

Models & analysis

n/a	Involved in the study
☐	☐ Functional and/or effective connectivity
☐	☐ Graph analysis
☐	☐ Multivariate modeling or predictive analysis

Functional and/or effective connectivity

na

Graph analysis

na

Multivariate modeling and predictive analysis

na
