# Supplementary material for: Accelerated nitrogen cycling on Mediterranean seagrass leaves at volcanic CO2 vents
Source: Commun Biol. 2024 Mar 19;7:341. doi: 10.1038/s42003-024-06011-0 (PMC11254932; doi:10.1038/s42003-024-06011-0)
Supplement: Supplementary file 1 — Peer Review File [file 42003_2024_6011_MOESM1_ESM.pdf]

nature portfolio

Peer Review File

Accelerated Nitrogen Cycling on Mediterranean seagrass leaves at volcanic CO2 vents

Open Access This file is licensed under a Creative Commons Attribution 4.0 International License, which permits use, sharing, adaptation, distribution and reproduction in any medium or format, as long as you give appropriate credit to the original author(s) and the source, provide a link to the Creative Commons license, and indicate if changes were made. In the cases where the authors are anonymous, such as is the case for the reports of anonymous peer reviewers, author attribution should be to 'Anonymous Referee' followed by a clear attribution to the source work. The images or other third party material in this file are included in the article's Creative Commons license, unless indicated otherwise in a credit line to the material. If material is not included in the article's Creative Commons license and your intended use is not permitted by statutory regulation or exceeds the permitted use, you will need to obtain permission directly from the copyright holder. To view a copy of this license, visit http://creativecommons.org/licenses/by/4.0/.

Reviewers' comments:

Reviewer #1 (Remarks to the Author):

Dear Berlinghof et al.,

This was an exciting paper to read because of the use of the natural CO_2 vents to answer questions related to the future of OA in the seagrass holobiont. They present the intriguing finding that there is no difference in microbial community between vent/no-vent but there are differences within the nitrogen cycling as demonstrated by the experiments. The flow of the paper was clear and the results were presented well, with some caveats. The methodology section could do with some clarifications which will hopefully with help the reader understand the experimental context better. The last section, albeit a necessary and logical progression, seemed to appear out of thin air with numbers coming from unknown hidden regions of the text. This broke the flow of any otherwise well-written research paper. I do see this as being of value to the scientific community and of the caliber of this journal. However, the fundamental claim of the paper begs a modicum of constructive scrutiny which is a necessary feature of the peer review process.

The Scrutiny:

Clarification in the methods would be helpful especially given the number of categories tested for. It's very evident in the methods and the results that the authors are looking for the effect of light/dark, CO_2 enrichment/non-CO_2 enrichment and epiphyte/non-epiphyte. This makes for 8 different combinations for which it is difficult to find a useful control for each comparison set. In the Nitrogen Fixation experiment, the authors controlled for the CO_2 enrichment and light-dark (I assume…) but included epiphytes in the control. In the Dinitrification/Annamox and in the nitrification experiments, the controls controlled for epiphyte/no epiphyte, CO_2 and light dark environments with the goal of assessing background microbial activity. However, in these controls, the authors also did not include the NH_4^+ nor the NO_3^- enrichments in these controls which means that the background microbes in the 'control' aren't engaging with the same nutrient environment as those in the enrichment experiments. This naturally has knock on effects on how the controls influence the analysis of the data. A data table with columns as experiments and rows as factors tested might be a useful endeavor.

The results are interesting but may be overstated, in part related to the nutrient-enrichments. In each of the enrichments, a key factor to consider is that these are nutrient enrichments. NO_3^- and NH_4^+ concentrations (N_2 N-fixation concentrations were not written) are almost an order of magnitude greater than ambient conditions. Therefore, statements commenting on the differences between CO_2 enrichments reflect these unrealistically nitrogen loaded conditions rather than ambient nutrient conditions. These statements, however, hold weight primarily due to the experiment being successful at discerning relative differences between vent/non-vent conditions. By elevating the nutrient concentrations so much, the experiment simply boosts the signal allowing the authors to discern the differences in conditions while in reality (ambient conditions), the difference in signal might be too small

to make too large of a difference to the holobiont.

The Results section "Phyllosphere N cycling contributes to the holobiont N demand" is a necessary one as it takes the authors' experimental data and extrapolates it to the rest of the year, seeking comparisons across the relevant literature. However, Fig. 4 and the numbers used rather appear out of nowhere. If these are all derived from the experiments, there needs to be a line in the methods sections explaining this – which could possibly include how the authors had included NCP results from the other Berlingof et al. paper. It's a well known fact that nitrogen availability influences carbon-cycling and the authors take on the task of drawing the lines between the two. However, the numbers again are called into question due to the points I've raised above. Do the numbers come from the experiments? If so, do these numbers account for the fact that the experiment was held in unrealistically nutrient rich waters while the NCP values were taken a research paper from waters/experiments not nutrient-enriched?

Taking all this into account. The final and most intriguing claim of the paper is that in unaltered conditions, the microbial communities of vent/no-vent are no different. But in the experiments Nitrogen cycling is different. From the experimental set-up, I'm inclined to believe that this is due to the unrealistic nitrogen loads placed on the microbial communities. I would expect that if these naturally occurring microbial communities in the vent/no-vent environment were exposed to these highly elevated NO_3^- and NH_4^+ conditions, they too would change. This needs to be considered especially while establishing a synthesis between the two components (experimental and microbial aspects) of this research paper.

Best,
Khashiff Miranda

Line by Line:
L54-56: In Fig. 1c of Berlingof et al. 2022, both inside and outside vent seagrass communities from the same island community seem to show that both have at least a 35% epiphyte cover with differences in community composition. This claim forms the basis of the experiment of this research paper. However, this paper focuses on epiphytes vs no epiphytes. Why? Why not differences in epiphyte-impacts? Berlingof et al. 2022 doesn't seem to say the future would include epiphytes. Would the authors expect different results in this research context if the epiphytes were bryozoans vs algae. Here is also where it needs to be specified what is the identity of epiphytes. Bryozoans vs hydrozoans vs algae will play entirely different roles in nutrient cycling.

L84: "besides N_2 fixation" why would you expect N_2 fixation be a big feature of the phyllosphere?

L104: Replicate numbers are confusing. Here it's n=4, in methods section, it indicates that sometimes 3 or 4 vials were sampled.

L114: Very cool.

L118: Specify whether these root tissues were found in vented areas.

L125: ‘significant but only marginal’? Marginal in terms of the amount of PNR?

L127: light vs dark, light with no epiphytes sees more than dark with no epiphytes. Suggests that the epiphytes are potential competitors for NH4 in nitrification?

L150: add a space between super script and reference number.

Section: Distinct microbial communities contribute to seagrass phyllosphere N cycling
This section is simply and does a good job of relating this research to the wealth of microbial research available.

L250: Microbial community is stable but relative abundances may be different, while the function of each community member might differ too.

L259: This entire section works well with the goal of the paper but has nothing explaining the methods in the Methods section. The numbers in this section seemingly appear out of nowhere. If they are based off the experiments, note the point I raised in the beginning of the review about comparing the experiments to the ambient nutrient environment.

L267: How was Fig. 4 even generated?!

L288: Who measured these spring values? References?

L292: What are projected values of OA in the Mediterranean in the future? Could be useful to put in the introduction/would strengthen your assertions if the vent pH resembles that of future projections.

L315: Why select only the leaves with homogenized epiphyte cover? Would you expect there to be a difference in the microbial community/photosystem between leaves with no epiphyte cover vs leaves with epiphyte cover that have had the epiphytes removed? Removing the epiphyte cover from these leaves surely would impact the microbial community naturally occurring on these leaves leaving these sections relatively sterile compared to naturally occurring leaves with no epiphytic cover.

L367: what were ambient N2 concentrations/ did your enrichments vastly increase these concentrations?

L371: Specify how many leaves in each vial. Did tissue pooling occur?

L374: I’m not too sure if these may be considered as ‘controls’ if they considered leaves with epiphytes. Perhaps this would be the control for the enriched water containing leaves with epiphytes, but it would not be an appropriate control for leaves without epiphytes. Had you conducted this by any chance with just the leaves (I’d use that data)? If not, I’d indicate perhaps in the methods section that the authors had

considered the presence of epiphytes negligible to gross 15N production in the unenriched water.

L375: For the light and dark incubations, what were the replicates? It seems like the split of epiphyte/no epiphyte was n=3, n=4. But after that as well these were split into further subreplicates for light/dark experiments? It seems like for every experiment, there should be three categories: light/dark, epiphyte/no epiphyte, vent/ambient. This should make 8.

L377: Given the small capacity of the 24ml vial, would the authors expect that opening the samples would influence the oxygen environment? Did the O2 sampling entail the firesting probe or the dot sensors?

L382: Post incubations, I take it the epiphytes were removed by scalpel from the leaves with epiphytes for downstream tissue analysis?

L410: Was there any quantification of amount of epiphytic growth in terms of weight? Again epiphyte identity will be useful.

L415: 15NH4 enrichment is just under a couple orders of magnitude greater than ambient conditions. 15NO3- conditions were an order of magnitude greater in enrichments vs ambient conditions.

L471: “filtered site water” specify site with/without vent

L472: Controls across the three experiments seem to be very different. L472 has only water as controls, attempting to control for microbial community activity in water.

Fig. 3: All leaf samples pooled vs all water samples?

Reviewer #2 (Remarks to the Author):

The manuscript by Berlinghof et al describes experiments on nitrogen cycling in seagrass leaves. The authors used Podidonia from two sites, a vent site with lower pH, simulating increased ocean acidification, and a 'control' site with ambient pH. They could show that most nitrogen cycling processes, including nitrogen fixation, were accelerated at the vent site. Additional 16S rRNA amplicon sequencing shows similar communities at both sites, but slight increases in taxa that are presumably associated with different processes in the N cycle.

The manuscript is well written and the experiments are of high standard. Below, I list my main concerns:

1) It is unfortunate that only one site was chosen for each condition. There are probably many other physicochemical parameters that are different at the vent site and it would have been much more significant if multiple sites/meadows would have been tested.

2) While the authors discuss the holobiont and studies on microorganisms in the seagrass rhizosphere, here only small parts of shoots are being used. I would have prefered to see experiments with whole plants as the results on leaves and roots might differ significantly. Additionally, the metabolism of the whole plant would presumably have an impact on these studies, too.

3) The authors mention that they used Data2 and ASV tables, but the figures are based on phyla and order levels. This is not very helpful as these higher taxonomic levels are too divers.

Answer to Reviewers' comments:

Reviewer #1:

Dear Berlinghof et al.,

This was an exciting paper to read because of the use of the natural CO2 vents to answer questions related to the future of OA in the seagrass holobiont. They present the intriguing finding that there is no difference in microbial community between vent/no-vent but there are differences within the nitrogen cycling as demonstrated by the experiments. The flow of the paper was clear and the results were presented well, with some caveats. The methodology section could do with some clarifications which will hopefully with help the reader understand the experimental context better. The last section, albeit a necessary and logical progression, seemed to appear out of thin air with numbers coming from unknown hidden regions of the text. This broke the flow of any otherwise well-written research paper. I do see this as being of value to the scientific community and of the caliber of this journal. However, the fundamental claim of the paper begs a modicum of constructive scrutiny which is a necessary feature of the peer review process.

Answer*: We appreciate the reviewer's constructive comments and have carefully considered them to enhance and clarify the manuscript. Below is a detailed point-by-point response addressing all of your comments.*

The Scrutiny:

Clarification in the methods would be helpful especially given the number of categories tested for. It's very evident in the methods and the results that the authors are looking for the effect of light/dark, CO2 enrichment/non-CO2 enrichment and epiphyte/non-epiphyte. This makes for 8 different combinations for which it is difficult to find a useful control for each comparison set.

Answer*: We thank the reviewer for pointing that out. We understand that our experimental setup was relatively complex. Therefore, we have now included an overview scheme of the incubation experiment in the Supplementary Material. Information about the different controls is provided in the answers below. Please find the overview scheme also here below for ease of review:*

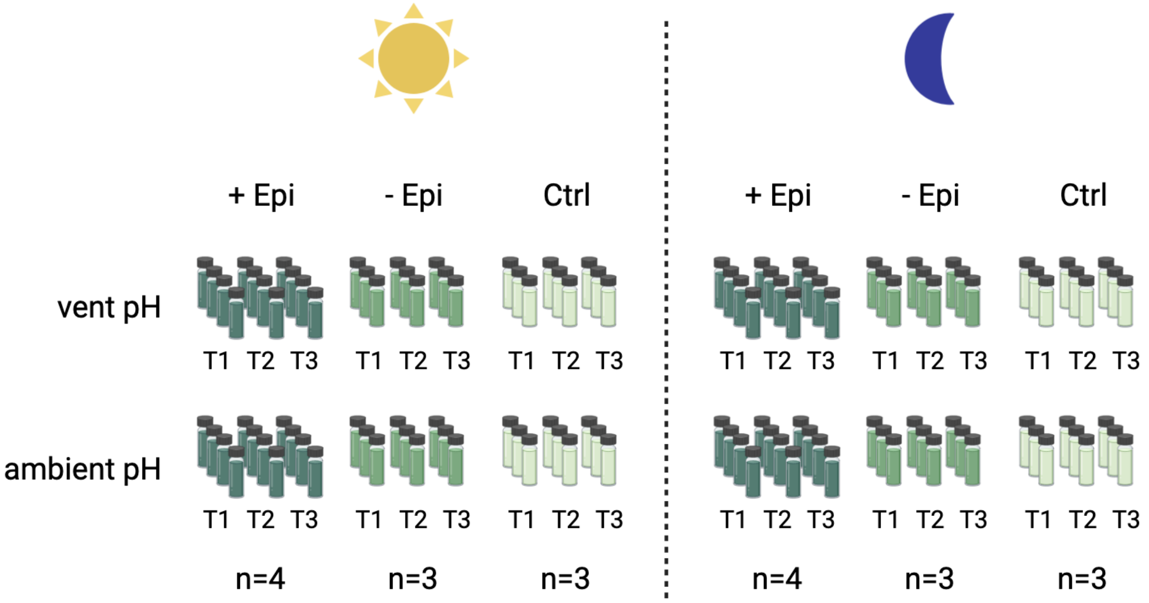

Suppl. Figure 1*. Experimental design of the three isotopic tracer incubation experiments with light and dark incubations, leaves from vent and ambient pH, with epiphytes present or removed, as well as controls. T1, 2, and 3 represent samples that were opened at different timepoints.*

In the Nitrogen Fixation experiment, the authors controlled for the CO2 enrichment and light-dark (I assume…) but included epiphytes in the control.

Answer*: The experiment aimed to detect N_2 fixation associated with epiphytes. Incubation without epiphytes + $^{15}N_2$ served as a control for nonspecific binding and abiotic incorporation by the leaves. Incubation with epiphytes without $^{15}N_2$ (no label + biomass) served as control to check for potential variation in natural abundance of ^{15}N during the incubation both in the epiphytes and in the leaves. We have now clarified this better in the methods at lines 399-402: "Additionally, vials with 0.22 µm filtered, but unenriched site water containing leaves with epiphytes served as controls to account for potential variation in natural abundance of ^{15}N in epiphytes or leaves (n = 3, see also Suppl. Fig. 1 for the experimental design)."*

In the Denitrification/Annamox and in the nitrification experiments, the controls controlled for epiphyte/no epiphyte, CO2 and light dark environments with the goal of assessing background microbial activity.
However, in these controls, the authors also did not include the NH4+ nor the NO3- enrichments in these controls which means that the background microbes in the 'control' aren't engaging with the same nutrient environment as those in the enrichment experiments. This naturally has knock on effects on how the controls influence the analysis of the data.

Answer*: Unfortunately, this was an oversight in the methods description of the original version of the manuscript. We have now rectified and can clarify here that the controls received the same nutrient enrichment as the experimental groups (i.e. with the $^{15}NH_4^+$ or $^{15}NO_3^-$ tracers), excluding the addition of seagrass leaves (i.e., "no biomass + label" controls), thereby controlling for potential residual microbial activity in the 0.2µm-filtered seawater*

during the nitrification and denitrification/anammox experiments. This is now also clarified in the text (lines 447-449 and 500-502).

A data table with columns as experiments and rows as factors tested might be a useful endeavor.

***Answer**: See comment above, overview included as Suppl. Fig. 1.*

The results are interesting but may be overstated, in part related to the nutrient-enrichments. In each of the enrichments, a key factor to consider is that these are nutrient enrichments. NO3- and NH4+ concentrations (N2 N-fixation concentrations were not written) are almost an order of magnitude greater than ambient conditions. Therefore, statements commenting on the differences between CO2 enrichments reflect these unrealistically nitrogen loaded conditions rather than ambient nutrient conditions. These statements, however, hold weight primarily due to the experiment being successful at discerning relative differences between vent/non-vent conditions. By elevating the nutrient concentrations so much, the experiment simply boosts the signal allowing the authors to discern the differences in conditions while in reality (ambient conditions), the difference in signal might be too small to make too large of a difference to the holobiont.

***Answer**: We acknowledge the effects of nutrient enrichment, and indeed defined the process rates that we measured in our experiments as "<u>potential</u>" denitrification/anammox and nitrification rates throughout the manuscript. Similar measurements are routinely performed in short-term incubations to measure assumed maximum rates of nitrate, nitrite or N_2 production under optimal, non-substrate-limiting conditions. While the method has inherent limitations, it is used to determine the "potential" or maximum rate of a functional process in environmental samples.*
In the denitrification experiment, [NO_3^-] was increased from natural 1.94 ± 1.11 µM to 9.59 ± 1.71 µM in enriched incubations. While this is clearly an enrichment in background [NO_3^-], this is not far off of natural environmental variability at the site (see for example Garrard, 2013. The effect of ocean acidification on plant-animal interactions in a Posidonia oceanica meadow, https://doi.org/10.21954/ou.ro.0000d5ba). Given that most microorganisms adapted to oligotrophic waters usually have their affinity for nitrate in the micromolar range (Algar and Vallino, 2014. Predicting microbial nitrate reduction pathways in coastal sediments. Aquat Microb Ecol, Vol. 71: 223–238), they should be able to rapidly adapt to a low micromolar concentration change.
For the nitrification experiment [NH_4^+] was increased from 0.65 ± 0.07 µM to 20.66 ± 2.35 µM. This is a larger concentration difference. This was chosen to maximize our opportunity to detect a signal. We found potential nitrification rates only at vent pH but point out in the manuscript that these potential rates were very low and therefore contributed little to the overall N budget of the P. oceanica phyllosphere. However, since we found specific nitrifying bacteria and archaea in the plant-associated microbial community, we still think that these results are of interest and worth reporting.

The Results section "Phyllosphere N cycling contributes to the holobiont N demand" is a necessary one as it takes the authors' experimental data and extrapolates it to the rest of the year, seeking comparisons across the relevant literature. However, Fig. 4 and the numbers

used rather appear out of nowhere. If these are all derived from the experiments, there needs to be a line in the methods sections explaining this – which could possibly include how the authors had included NCP results from the other Berlinghof et al. paper. It's a well known fact that nitrogen availability influences carbon-cycling and the authors take on the task of drawing the lines between the two. However, the numbers again are called into question due to the points I've raised above. Do the numbers come from the experiments? If so, do these numbers account for the fact that the experiment was held in unrealistically nutrient rich waters while the NCP values were taken a research paper from waters/experiments not nutrient-enriched?

***Answer**: We agree that this section needs to be better explained and linked to our methods. Therefore, we included the paragraph "Holobiont N demand calculations" in the methods section with information about how we obtained the numbers of Fig. 4 (lines 506-515). Please find it here below for ease of review:*
"Holobiont N demand calculations
To calculate daily metabolic rates of plant and epiphyte-mediated N cycling processes, we integrated rates of N_2 fixation, nitrification potential, N loss (denitrification and anammox), NO_3^- , and NH_4^+ uptake in the light and dark incubations assuming a daily 12:12 h light/dark cycle. This way we obtained daily rates in mmol N $m^{-2}_{leaf\ area}$ d^{-1} at vent and ambient pH. We further used net community productivity (NCP) from Berlinghof et al.[12] (using a photosynthetic quotient of 1), C:N ratios (Suppl. Fig. 7), average leaf density and dry weight per leaf at the ambient and vent site (Suppl. Table 5) to calculate the potential percentage of daily primary production of the seagrass holobiont (plant + epiphytes) that can be supported by leaf-associated N_2 fixation."

Taking all this into account. The final and most intriguing claim of the paper is that in unaltered conditions, the microbial communities of vent/no-vent are no different. But in the experiments Nitrogen cycling is different. From the experimental set-up, I'm inclined to believe that this is due to the unrealistic nitrogen loads placed on the microbial communities. I would expect that if these naturally occurring microbial communities in the vent/no-vent environment were exposed to these highly elevated NO3- and NH4+ conditions, they too would change. This needs to be considered especially while establishing a synthesis between the two components (experimental and microbial aspects) of this research paper.

***Answer**: As stated above, nitrate concentrations were not elevated much beyond the range reported for the sites. Furthermore, both vent and ambient samples were exposed to the same experimental concentrations, yet, alternatively to the null hypothesis (i.e., no change) or the reviewer's hypothesis stated above (i.e., both would change), we observed significant differences in the N-fluxes between the sites. Please note that the microbial community was sampled directly from the natural site and independently from the incubation experiments. The similarity between the vent and ambient microbial communities thus suggests similar seed communities for the experimental incubations.*

Reviewer 1 comments line by line:

L54-56: In Fig. 1c of Berlinghof et al. 2022, both inside and outside vent seagrass communities from the same island community seem to show that both have at least a 35% epiphyte cover

with differences in community composition. This claim forms the basis of the experiment of this research paper. However, this paper focuses on epiphytes vs no epiphytes. Why? Why not differences in epiphyte-impacts? Berlinghof et al. 2022 doesn't seem to say the future would include epiphytes. Would the authors expect different results in this research context if the epiphytes were bryozoans vs algae. Here is also where it needs to be specified what is the identity of epiphytes. Bryozoans vs hydrozoans vs algae will play entirely different roles in nutrient cycling.
Answer*: The reason why we tested epiphyte vs. no epiphyte was to investigate whether prokaryote-mediated N-cycling processes occur in the phyllosphere of the seagrass. In the Berlinghof et al. 2022 paper the focus was on differences in the larger epiphytic community (algae, bryozoans, etc.), while here we specifically investigated prokaryotes and their role in N cycling. Differences in these processes due to different pH regimes will also affect larger epiphytes, and this is certainly worth further research and experiments. However, this was not focus of the present study.*

L84: "besides N2 fixation" why would you expect N2 fixation be a big feature of the phyllosphere?
Answer*: There is literature on N_2 fixation in the phyllosphere of P. oceanica, for example by Agawin et al. (2016) who found that this can significantly contribute to the overall N demand of the plant. We clarified this with relevant references in the text at lines 89-90: "Phyllospheric N_2 fixation can significantly contribute to the N demand of P. oceanica and to the N budget in the Mediterranean Sea [28,29]."*

L104: Replicate numbers are confusing. Here it's n=4, in methods section, it indicates that sometimes 3 or 4 vials were sampled.
Answer*: Here only epiphytic ^{15}N incorporation is shown. n was always =4 in incubations with epiphytes present. This should now be clarified in Suppl. Fig 1.*

L114: Very cool.
Answer*: Thank you!*

L118: Specify whether these root tissues were found in vented areas.
Answer*: Amended in line 125: "... measured by root symbionts of P. oceanica under ambient pH."*

L125: 'significant but only marginal'? Marginal in terms of the amount of PNR?
Answer*: Correct, we now clarified this in the text, lines 134-135: "However, these rates were only marginal in terms of the amount of PNR."*

L127: light vs dark, light with no epiphytes sees more than dark with no epiphytes. Suggests that the epiphytes are potential competitors for NH4 in nitrification?
Answer*: The results presented here show only rates in incubations where epiphytes were present. We have seen no nitrification potential in incubations where epiphytes were removed.*

L150: add a space between super script and reference number.
Answer*: Amended.*

Section: Distinct microbial communities contribute to seagrass phyllosphere N cycling. This section does a good job of relating this research to the wealth of microbial research available.
Answer*: Thank you!*

L250: Microbial community is stable but relative abundances may be different, while the function of each community member might differ too.
Answer*: Relative abundances were tested to be not different between vent and ambient communities (Suppl. Fig 6 and Suppl. Table 1). However, we acknowledge that the level of investigation (using 16s amplicon sequencing) is coarse and that subtle differences for specific microbial populations may exist. In line with the reviewer's comment, in the discussion we argue that differences in the investigated N cycling processes point towards a functionally (rather than structurally) different microbial community between inside and outside the vents (see lines 276-279).*

L259: This entire section works well with the goal of the paper but has nothing explaining the methods in the Methods section. The numbers in this section seemingly appear out of nowhere. If they are based off the experiments, note the point I raised in the beginning of the review about comparing the experiments to the ambient nutrient environment.
Answer*: We agree that a methodological explanation is needed, and we now added the paragraph "Holobiont N demand calculations" in the methods section (lines 514-523) with information about how we obtained the numbers reported in Fig. 4.*

L267: How was Fig. 4 even generated?!
Answer*: Thank you, see our comment above.*

L288: Who measured these spring values? References?
Answer*: The spring values were also measured by Lepoint et al. We now added the reference in the text (line 307).*

L292: What are projected values of OA in the Mediterranean in the future? Could be useful to put in the introduction/would strengthen your assertions if the vent pH resembles that of future projections.
Answer*: We agree that this can provide more context, and added predicted pH values for the Mediterranean Sea to the introduction, with a relevant citation, see lines 48-51: "The pH of the Mediterranean Sea in the Western basin is predicted to decrease between 0.245 under the most optimistic scenario of the "Special Report: Emissions Scenarios" (SRES) of the IPCC (2007) and 0.462 units under the most pessimistic SRES scenario."*

L315: Why select only the leaves with homogenized epiphyte cover? Would you expect there to be a difference in the microbial community/photosystem between leaves with no epiphyte cover vs leaves with epiphyte cover that have had the epiphytes removed? Removing the epiphyte cover from these leaves surely would impact the microbial community naturally occurring on these leaves leaving these sections relatively sterile compared to naturally occurring leaves with no epiphytic cover.
Answer*: We selected for homogenous coverage of leaf epiphytes for better comparability, because we wanted to avoid the confounding factor of different epiphytic loads. The removal*

of the epiphytes served as control for presence/absence of the associated prokaryotic communities, as rightly pointed out by the reviewer.

L367: what were ambient N2 concentrations/ did your enrichments vastly increase these concentrations?
***Answer**: Dissolved N_2 gas has concentrations of over 400 µmol L^{-1} in sea water. While the absolute concentration depends on temperature and salinity (which was the same for enriched vials vs controls) and the $^{15}N_2$-enriched seawater addition method does not significantly affect the overall dissolved N_2 gas concentration, the method significantly modifies the isotopic ratio of $^{15}N_2$ over $^{14}N_2$, which allows to measure incorporation of the heavier isotope into biomass. In particular, "with our approach, we achieved an enrichment of ~16.0 atom% ^{15}N in the incubation vials" (see lines 429-430); this is nearly 50-fold increase in ^{15}N but only 1.15-fold increase in N_2 concentration.*

L371: Specify how many leaves in each vial. Did tissue pooling occur?
***Answer**: It was one leaf per vial, we now clarified this in the method section (lines 397-399).*

L374: I'm not too sure if these may be considered as 'controls' if they considered leaves with epiphytes. Perhaps this would be the control for the enriched water containing leaves with epiphytes, but it would not be an appropriate control for leaves without epiphytes. Had you conducted this by any chance with just the leaves (I'd use that data)? If not, I'd indicate perhaps in the methods section that the authors had considered the presence of epiphytes negligible to gross 15N production in the unenriched water.
***Answer**: With the "no label + biomass" control we wanted to exclude any potential variation in natural abundance of ^{15}N during the incubation both in the epiphytes and in the leaves. We now explained this in the method section (lines 399-402).*

L375: For the light and dark incubations, what were the replicates? It seems like the split of epiphyte/no epiphyte was n=3, n=4. But after that as well these were split into further subreplicates for light/dark experiments? It seems like for every experiment, there should be three categories: light/dark, epiphyte/no epiphyte, vent/ambient. This should make 8.
***Answer**: There were 4 replicates for leaves with epiphytes and 3 for leaves without epiphytes, in both the light and dark incubations. A scheme of the experimental design was added to the supplementary material (Suppl. Fig. 1).*

L377: Given the small capacity of the 24ml vial, would the authors expect that opening the samples would influence the oxygen environment? Did the O2 sampling entail the firesting probe or the dot sensors?
***Answer**: Oxygen was measured without opening the vials using the firesting dot sensors. This is now better clarified in the text (lines 405-408).*

L382: Post incubations, I take it the epiphytes were removed by scalpel from the leaves with epiphytes for downstream tissue analysis?
***Answer**: Yes, this is now better clarified in the text (lines 409-410).*

L410: Was there any quantification of amount of epiphytic growth in terms of weight? Again epiphyte identity will be useful.

Answer*: Yes, epiphytes were separated and weighed as clarified now in lines 409-410. The community composition besides the microbial community is discussed in Berlinghof et al. (2022), but since the focus of this paper is on the microbial community, we did not include it here, but rather cite our previous study.*

L415: $^{15}NH_4^+$ enrichment is just under a couple orders of magnitude greater than ambient conditions. $^{15}NO_3^-$ conditions were an order of magnitude greater in enrichments vs ambient conditions.
Answer*: Yes, this was the case, because ambient NH_4^+ and NO_3^- concentrations were not known for this specific site at the time of the experiment. We used ambient concentrations from previous measurements and estimated the enrichment concentrations needed to effectively quantify potential nitrification and denitrification/anammox. The upshot is that the NH_4^+ experiment was more sensitive to measure trace levels of "potential" ammonia oxidation rates.*

L471: "filtered site water" specify site with/without vent
Answer*: Controls for both vent and ambient pH were included. This is now clarified in the text (lines 500-502).*

L472: Controls across the three experiments seem to be very different. L472 has only water as controls, attempting to control for microbial community activity in water.
Yes, the controls were different for the N_2 fixation experiment and the others because we controlled for different factors (other than the ones being tested). There was an oversight in the original version of our manuscript, as controls for the nitrification and denitrification/anammox experiments were wrongly described as only seawater without label, which was not the case. The controls received the same nutrient enrichment as the experimental groups, but neither leaves nor epiphytes (no biomass + label), to control for potential residual microbial activity in the filtered seawater. Conversely, in the N_2 fixation experiment, incubation with epiphytes without $^{15}N_2$ (no label + biomass) served as control to check for potential variation in natural abundance of ^{15}N during the incubation both in the epiphytes and in the leaves. This is now clarified in the text (lines 399-402 and 500-502) and in our previous answers here.

Fig. 3: All leaf samples pooled vs all water samples?
Answer*: Correct, we now clarified this in the figure legend and the method section (line 233 + 387-388).*

Reviewer #2:

The manuscript by Berlinghof et al describes experiments on nitrogen cycling in seagrass leaves. The authors used Posidonia from two sites, a vent site with lower pH, simulating increased ocean acidification, and a 'control' site with ambient pH. They could show that most nitrogen cycling processes, including nitrogen fixation, were accelerated at the vent site. Additional 16S rRNA amplicon sequencing shows similar communities at both sites, but slight increases in taxa that are presumably associated with different processes in the N cycle.

The manuscript is well written, and the experiments are of high standard.

***Answer**: We express our gratitude to the reviewer for their efforts in reviewing the manuscript and providing constructive comments. We have given careful consideration to each comment and endeavored to comprehensively address them in our point-by-point response below.*

Below, I list my main concerns:

1) It is unfortunate that only one site was chosen for each condition. There are probably many other physicochemical parameters that are different at the vent site, and it would have been much more significant if multiple sites/meadows would have been tested.

***Answer**: We acknowledge that our work would have benefitted from a comparison between a larger number of study sites. However, managing multiple sites would have required a considerably larger research team and more resources, which were beyond the scope of our project. Unfortunately, not many sites are available with comparable levels of CO_2 venting from the seafloor, comparable depth, light and hydrodynamics. Therefore, expanding the number of locations would have increased confounding factors which we would have had to control for, potentially affecting the reliability and consistency of our data or considerably expanding the scope of the project beyond our possibilities. We now included this information briefly in lines 330-333:*
"We restricted our study locations to these sites, because not many vent sites have comparable levels of CO_2, depth, light, and hydrodynamics. Increasing the number of locations would have increased confounding factors, potentially affecting the reliability and consistency of our data."

2) While the authors discuss the holobiont and studies on microorganisms in the seagrass rhizosphere, here only small parts of shoots are being used. I would have preferred to see experiments with whole plants as the results on leaves and roots might differ significantly. Additionally, the metabolism of the whole plant would presumably have an impact on these studies, too.

***Answer**: Our work aims to fill knowledge gaps specifically about prokaryotic N cycling processes in the phyllosphere, hence the choice to use leaves and not the whole plant. Additionally, while studies exploring processes at the level of the whole plant are important and much needed, focusing on the leaves allowed us to control for the community composition within the phyllosphere, which is exposed to oxygen-rich seawaters. On the contrary, having also root tissues in our incubations would have complicated both the experimental setup and the interpretation of results, as contrasting processes can occur between the (mainly) oxidized aboveground phyllosphere and the (mainly) reduced belowground rhizosphere. We now included this information briefly in lines 343-346:*
"Focusing on the leaves allowed us to control for the community composition within the phyllosphere exposed to oxygen-rich seawaters and avoiding contrasting processes occurring between the mainly oxidized aboveground phyllosphere and the mainly reduced belowground rhizosphere."

3) The authors mention that they used Data2 and ASV tables, but the figures are based on phyla and order levels. This is not very helpful as these higher taxonomic levels are too divers.

Answer*: Thank you for the observation. As the reviewer pointed out, we are aware that the presentation of prokaryotic communities at high taxonomic levels may reduce the possibility of highlighting differences between communities. However, considering the large number of ASVs (n=2285) obtained by the dada2 analysis, this choice was considered necessary for the sake of simplifying and summarizing the findings on microbial community analyses. Nevertheless, the PCoA, PERMANOVA and differential abundance analysis are based on ASV-level data. To address the Reviewer's suggestion, we have added additional descriptions and references of the most relevant groups we found at genus level or at the best taxonomic resolution we could achieve with our dataset (see lines 181-185, 189-192). Additionally, figures 2 and 3 now reflect the results also at genus level.*

Reviewers' comments:

Reviewer #2 (Remarks to the Author):

The authors have reworked their manuscript on how differential CO_2 environments may influence N-cycling in the seagrass microbiome. It's an important study in that it provides hard numbers relating OA to various N-cycling processes. It is also clear that the authors addressed the reviewers comments in a satisfactory manner, however, there are still a few points that would help the flow of the paper in terms of ordering the sections and paying close attention to the language relating to microbial/macro-epiphytes which makes the paper a rather complicated read for the readers, especially given the complex nature of the experimental set up.

At the time of first submission, it was a novel research subject however, the recent publication by Pfister et al. 2023 in Nature does do a similar study relating the vent environment to N-cycling and subsequently C-production (with that research team referencing this paper in review). The novelty of this paper is thus the emphasis on differential nitrogen cycling despite similar microbial communities as well as the inclusion of the role of epiphytes.

Detailed comments.

Abstract: Could put in a reference to epiphytes.

L100: specify eukaryotic/macro-epiphytes. Otherwise can get confused with the microbial epiphytes.

Fig1 Caption: does (c) have epiphytes for $^{30}N_2$ production? If so then all of the graphs have epiphytes and it should be reflected in the boldface section of the caption or in the title of the graph. If not, wherever leaf sections had no epiphytes, that should be reflected. Important to situate your readers with this caption.

Fig1: N-fixation happening primarily during photoperiod and not at night? Very peculiar. Any explanations?
Add asterisks to point out significance values.

L115-128. Need to specify microbial epiphytes/macro-epiphytes. If not, it gets awfully confusing for the reader.

L129. Writing 'fixed N … can be recycled … by nitrification' implies that there is a connecting pathway between N-fixation to the nitrification. Is that what this paragraph implies (tied into the point below)? The ^{15}N-NH_4 enrichment looks to be an external source of N and the experimental situation doesn't lead to this conclusion. Better to cut the first few words and write "NH_4^+ can be recycled and converted into NO_3^- by nitrification" but that is commonly known and not a good introductory line. Workshop this.

L129-145: If I have this paragraph correct, the authors are trying to assert that from Fig. 1a, CO_2 vents increase NH_4 availability through N-fixing. This in turn creates enough NH_4 for nitrification to be present. The conclusion thus drawn is that vents create a good enough environment for nitrifiers to live? If so, this is really interesting!
In this situation, the presence of the epiphytes remain interesting to me. If the epiphytic community is indeed different between the vent/no-vent sites, then the type of metabolisms too would be different between the two which could consequently influence the NH_4 environment. Could this be a

confounding variable in drawing this conclusion?

L134-135: 'marginal in terms of the amount of PNR'. Where?

L160-161: 'no3 loss due to denitrification… activity was three orders of magnitud lower' where is this value coming from?

L166: My preference would be to place the 'distinct microbial communities' as the first section of the Results and Discussion then you follow it up with 'Complete microbial N cycling occurs in the P. oceanica phyllosphere' section. It would better introduce the microbial epiphytes and drive home the message about all the actors involved in this intricate nitrogenous dance. However, this is simply a stylistic suggestion.

Had not measured ambient pH. Was this controlled for pH in the experiments ? If ambient pH was different but for the experiment, they were placed across pHs, this would be a

Fig. 4:
- Epiphytes also might be able to uptake NH_4^+.
- "The arrow size indicates the magnitude of N cycling rates." As both thickness and length vary while some arrows are curved and others aren't, it is not clear how to visually compare arrows. To show magnitude, vary only one. Or simply have the schematic reflecting relative differences.
- This is the first time the reader gets to see the pH of ambient vs CO_2 vents. This needs to be included further up in the paper. Make life easier for your readers.

L297: Building off Agawin's point of allochthonous N sources, the values estimated in the Holobiont section relate to those measured in the lab setting. Do the authors expect these lab values to deviate greatly from reality given external nitrogen cycling processes/presence of animals that greatly contribute NH_4^+ to their ecosystems?

Reviewer #3 (Remarks to the Author):

The authors addressed all my concerns appropriately. The main issue with replication still stands, though. Maybe at least toning down that title '... seagrass leaves in a high CO_2 world' would be appropriate to match the fact that only one vent site and only one seagrass species were studied...

Reviewers' comments:

Reviewer #1 (Remarks to the Author):

The authors have reworked their manuscript on how differential CO2 environments may influence N-cycling in the seagrass microbiome. It's an important study in that it provides hard numbers relating OA to various N-cycling processes. It is also clear that the authors addressed the reviewers comments in a satisfactory manner, however, there are still a few points that would help the flow of the paper in terms of ordering the sections and paying close attention to the language relating to microbial/macro-epiphytes which makes the paper a rather complicated read for the readers, especially given the complex nature of the experimental set up.

At the time of first submission, it was a novel research subject however, the recent publication by Pfister et al. 2023 in Nature does do a similar study relating the vent environment to N-cycling and subsequently C-production (with that research team referencing this paper in review). The novelty of this paper is thus the emphasis on differential nitrogen cycling despite similar microbial communities as well as the inclusion of the role of epiphytes.

Answer: We sincerely appreciate Reviewer 1's detailed and constructive feedback on our manuscript. We have given careful consideration to your comments to enhance the overall clarity and flow of the paper.

Regarding the distinction between micro- and macro- epiphytes, we have made the decision not to differentiate between the two in this manuscript. Our method of scraping off the macro-epiphytes and biofilm with a scalpel was designed to effectively remove the majority of microbial epiphytes. Therefore, when we refer to seagrass epiphytes, we are encompassing both micro- and macro-epiphytes in our discussion. It is important to note that the nitrogen cycling processes highlighted in Fig. 1 are mediated by microorganisms and were observed only in the presence of epiphytes, emphasizing the role of the microbial community and confirming the effectiveness of our method to remove most microbial players from the leaves. This distinction is explicitly outlined in the presentation and discussion of the results related to the investigated nitrogen cycling processes, however we now also added an explanatory sentence in the methods, to clarify the above in our manuscript (L345-346): "Macro-epiphytes and biofilm were carefully removed from half of the seagrass leaves with a scalpel, ensuring the removal of the majority of microbial epiphytes and taking special care not to damage the plant tissue"

In our revised manuscript, we have also included a reference to the study by Pfister et al. (L76-78). While their research was conducted in a similar system (P. oceanica meadows under CO_2 vent and ambient pH), the focus of their study differed. They investigated the fate of organic nitrogen in the form of amino acids and its transfer to the seagrass via microbial ammonification. In contrast, our study delved into the cycling and transformation of inorganic nitrogen across various processes within the phyllosphere of P. oceanica, providing a more detailed exploration of the microbial community. We believe that these studies are distinct from one another, and our manuscript still offers unique and novel insights.

Detailed comments.

Abstract: Could put in a reference to epiphytes.
Answer: Added to abstract (L29): "… while the epiphytic microbial community structure remains largely unaffected".

L100: specify eukaryotic/macro-epiphytes. Otherwise, it can get confused with the microbial epiphytes.
Answer: Please see answer above why we decided to clarify this in the methods section, but to not discriminate micro- and macro-epiphytes throughout this manuscript.

Fig1 Caption: does (c) have epiphytes for 30N2 production? If so then all of the graphs have epiphytes and it should be reflected in the boldface section of the caption or in the title of the graph. If not, wherever leaf sections had no epiphytes, that should be reflected. Important to situate your readers with this caption.
Answer: Yes, all data shown in this figure is from incubations with epiphytes, while these processes where not detected when (micro- and macro-) epiphytes were removed. We now clarified this in the figure caption (L112).

Fig1: N-fixation happening primarily during photoperiod and not at night? Very peculiar. Any explanations?
Answer: Yes, we discuss this in L197-202: "Higher N_2 fixation rates under light conditions suggest a diazotrophic community dominated by species that can cope with O_2 production from daytime photosynthesis, which would otherwise irreversibly inhibit the enzyme nitrogenase. Among the genera that can sustain N_2 fixation in the light, the leaves from both pH regimes comprised sequences for Schizothrix (0.22% on leaves vs. 0.01% in the water column) and Trichodesmium (up to 0.5% on leaves vs. 0.002% in the water column)."

Add asterisks to point out significance values.
Answer: We added asterisks to the figure and a description for the figure legend (L115).

L115-128. Need to specify microbial epiphytes/macro-epiphytes. If not, it gets awfully confusing for the reader.
Answer: Please see answer above why we decided to clarify this in the methods section, but to not discriminate micro- and macro-epiphytes throughout this manuscript.

L129. Writing 'fixed N … can be recycled … by nitrification' implies that there is a connecting pathway between N-fixation to the nitrification. Is that what this paragraph implies (tied into the point below)? The 15N-NH4 enrichment looks to be an external source of N and the experimental situation doesn't lead to this conclusion. Better to cut the first few words and write "NH4+ can be recycled and converted into NO3- by nitrification" but that is commonly known and not a good introductory line. Workshop this.
Answer: Thank you for pointing this out. We agree that with our results we cannot draw this conclusion (see also next comment). We deleted the sentence and directly start with our findings.

L129-145: If I have this paragraph correct, the authors are trying to assert that from Fig. 1a, CO2 vents increase NH4 availability through N-fixing. This in turn creates enough NH4 for nitrification to be present. The conclusion thus drawn is that vents create a good enough environment for nitrifiers to live? If so, this is really interesting!
In this situation, the presence of the epiphytes remain interesting to me. If the epiphytic community is indeed different between the vent/no-vent sites, then the type of metabolisms too would be different between the two which could consequently influence the NH4 environment. Could this be a confounding variable in drawing this conclusion?
Answer: Thank you for pointing that out. We discuss the effects of ocean acidification on nitrification in L251-256. With our approach and results it is however not possible to draw such a conclusion and nitrification is generally negatively affected by OA. We mention however, that increasing CO_2 levels

could lead to higher autotrophic nitrification rates by reducing CO_2 limitation and that a diverse nitrifier community could adapt to a wider range of pH values.

L134-135: 'marginal in terms of the amount of PNR'. Where?
Answer: We changed the sentence for clarification (L134-135): "However, these rates were only marginal compared to the other N transformation processes."

L160-161: 'no3 loss due to denitrification… activity was three orders of magnitude lower' where is this value coming from?
Answer: This is a comparison between NO_3^- fluxes (Suppl. Fig. 5 c, d) and $^{29}N_2$ and $^{30}N_2$ production rates (Fig. 1c, d). We added the reference to Fig. 1c, d to the text for clarification (L 163).

L166: My preference would be to place the 'distinct microbial communities' as the first section of the Results and Discussion then you follow it up with 'Complete microbial N cycling occurs in the P. oceanica phyllosphere' section. It would better introduce the microbial epiphytes and drive home the message about all the actors involved in this intricate nitrogenous dance. However, this is simply a stylistic suggestion.
Answer: Thank you for your thoughtful suggestion regarding the organization of our manuscript. While we acknowledge that placing the section on 'distinct microbial communities' first in the Results and Discussion could be an alternative approach, we opted to present the biogeochemical data as the initial section, considering them as the core of our study. By presenting the biogeochemical data first, we aim to provide a solid context for readers to understand the results of these processes. To support the microbially mediated processes, we then introduce information about possible prokaryotic players involved in nitrogen cycling. This approach allows us to delve into the intricate details before zooming out to discuss the broader effects of ocean acidification and the ecological relevance of our findings. We appreciate your perspective and the opportunity to explain our rationale behind the organization of the manuscript.

Had not measured ambient pH. Was this controlled for pH in the experiments ? If ambient pH was different but for the experiment, they were placed across pHs, this would be a
Answer: We want to clarify that the pH values were monitored, and it was observed that they increased slightly during the light incubations, but remained stable in the dark incubations. We took measures to ensure that pH was adequately considered and controlled throughout the experiments. However, if there are specific aspects or concerns related to pH that you would like us to address or elaborate on, please provide additional details (the comment seemed incomplete here), and we will be more than happy to provide further clarification.

Fig. 4:
- Epiphytes also might be able to uptake NH4+.
Answer: We have found no significant effect of the presence of epiphytes on NH_4^+ uptake (Suppl. Fig 5.), indicating that NH_4^+uptake by epiphytes was negligible in our experiments. This is why we consider it primarily plant mediated. We now clarify this better in the text (L306-307).

- "The arrow size indicates the magnitude of N cycling rates." As both thickness and length vary while some arrows are curved and others aren't, it is not clear how to visually compare arrows. To show magnitude, vary only one. Or simply have the schematic reflecting relative differences.
Answer: Thank you for your observation. We changed the description in the legend to: "The arrow size indicates relative differences in N cycling rates." (L289).

- This is the first time the reader gets to see the pH of ambient vs CO2 vents. This needs to be included further up in the paper. Make life easier for your readers.
Answer: Information is provided at the beginning of the section (L243-244). We refer to this data also in the methods section (L334-335).

L297: Building off Agawin's point of allochthonous N sources, the values estimated in the Holobiont section relate to those measured in the lab setting. Do the authors expect these lab values to deviate greatly from reality given external nitrogen cycling processes/presence of animals that greatly contribute NH4+ to their ecosystems?
Answer: Thank you for bringing up an important consideration regarding the estimation of values in the Holobiont section, particularly in the context of lab settings and their potential deviation from real-world scenarios. In this section, our primary goal is on underscore the substantial contribution of N_2 fixation to the holobiont N demand, even under varying pH conditions. This observation is consistent with findings from other studies, such as Agawin et al. While we fully acknowledge the inherent limitations of laboratory experiments, recognizing that they can only serve as proxies for field processes, our focus in this section remains on phyllosphere N cycling. We do recognize the broader contributions of external nitrogen cycling processes, the influence of animals contributing NH_4^+, and the effects of seasonal and spatial variations (as mentioned in L 298-299) to seagrass ecosystem N cycling. However, the specific focus of our study is on the phyllosphere, and our experimental design allows us to provide insights into the dynamics of N cycling within this context.

Reviewer #2 (Remarks to the Author):

The authors addressed all my concerns appropriately. The main issue with replication still stands, though. Maybe at least toning down that title '... seagrass leaves in a high CO2 world' would be appropriate to match the fact that only one vent site and only one seagrass species were studied...

Answer: We sincerely appreciate Reviewer 2' positive feedback and constructive input. In response to your suggestion, we agree that modifying the title to better align with the scope of our study is appropriate. Therefore, we propose changing the title from "Accelerated Nitrogen Cycling on Seagrass Leaves in a High CO_2 World" to "Accelerated Nitrogen Cycling on Mediterranean seagrass leaves at volcanic CO_2 vents". This adjustment aims to reduce generalization and more accurately reflects the limitation in the number of study sites. We hope this modification addresses your concern.
